# Intentional coronary revascularization versus conservative therapy in patients after peripheral artery revascularization due to critical limb ischemia: the INCORPORATE trial

**DOI:** 10.1007/s00392-024-02487-2

**Published:** 2024-07-11

**Authors:** Gabor G Toth, Marianne Brodmann, Sadeek S Kanoun Schnur, Stanislaw Bartus, Mislav Vrsalovic, Oleg Krestianinov, Petr Kala, Jacek Bil, Robert Gil, Jan Kanovsky, Luigi Di Serafino, Luca Paolucci, Emanuele Barbato, Fabio Mangiacapra, Zoltan Ruzsa

**Affiliations:** 1 Department of Cardiology, University Heart Center Graz, Medical University Graz, Graz, Austria; 2 Division of Angiology, Department of Internal Medicine, Medical University Graz, Graz, Austria; 3 Department of Cardiology, Faculty of Medicine, Doctoral School of Clinical Medicine, University of Szeged, Szeged, Hungary; 4 Royal Cornwall Hospitals NHS Trust, Truro, UK; 5 II Dept of Cardiology, Medical College, Jagiellonian University, Krakow, Poland; 6 Department of Cardiology, University of Zagreb School of Medicine, Sestre Milosrdnice University Hospital Center, Zagreb, Croatia; 7 E. Meshalkin National Medical Research Center of the Ministry of Health of the Russian Federation, Novosibirsk, Russia; 8 University Hospital Brno and Medical Faculty of Masaryk University, Brno, Czech Republic; 9 Department of Invasive Cardiology, Centre of Postgraduate Medical Education, Warsaw, Poland; 10 National Medical Institute of the Internal Affairs and Administration Ministry, Warsaw, Poland; 11 Department of Advanced Biomedical Sciences, University of Naples Federico II, Naples, Italy; 12 Department of Medicine and Surgery, Research Unit of Cardiovascular Science, Università Campus Bio-Medico Di Roma and Fondazione Policlinico Universitario Campus Bio-Medico, Rome, Italy; 13 Department of Clinical and Molecular Medicine, Sapienza University of Rome, Rome, Italy

**Keywords:** Critical limb ischemia, Coronary artery disease, Fractional flow reserve, Coronary angiography

## Abstract

**Objectives:**

INCORPORATE trial was designed to evaluate whether default coronary-angiography (CA) and ischemia-targeted revascularization is superior compared to a conservative approach for patients with treated critical limb ischemia (CLI). Registered at clinicaltrials.gov (NCT03712644) on October 19, 2018.

**Background:**

Severe peripheral artery disease is associated with increased cardiovascular risk and poor outcomes.

**Methods:**

INCORPORATE was an open-label, prospective 1:1 randomized multicentric trial that recruited patients who had undergone successful CLI treatment. Patients were randomized to either a conservative or invasive approach regarding potential coronary artery disease (CAD). The conservative group received optimal medical therapy alone, while the invasive group had routine CA and fractional flow reserve-guided revascularization. The primary endpoint was myocardial infarction (MI) and 12-month mortality.

**Results:**

Due to COVID-19 pandemic burdens, recruitment was halted prematurely. One hundred eighty-five patients were enrolled. Baseline cardiac symptoms were scarce with 92% being asymptomatic. Eighty-nine patients were randomized to the invasive approach of whom 73 underwent CA. Thirty-four percent had functional single-vessel disease, 26% had functional multi-vessel disease, and 90% achieved complete revascularization. Conservative and invasive groups had similar incidences of death and MI at 1 year (11% vs 10%; hazard ratio 1.21 [0.49–2.98]). Major adverse cardiac and cerebrovascular events (MACCE) trended for hazard in the Conservative group (20 vs 10%; hazard ratio 1.94 [0.90–4.19]). In the per-protocol analysis, the primary endpoint remained insignificantly different (11% vs 7%; hazard ratio 2.01 [0.72–5.57]), but the conservative approach had a higher MACCE risk (20% vs 7%; hazard ratio 2.88 [1.24–6.68]).

**Conclusion:**

This trial found no significant difference in the primary endpoint but observed a trend of higher MACCE in the conservative arm.

**Graphical Abstract:**

A graphical abstract illustrating the key highlights of the design and comparisons

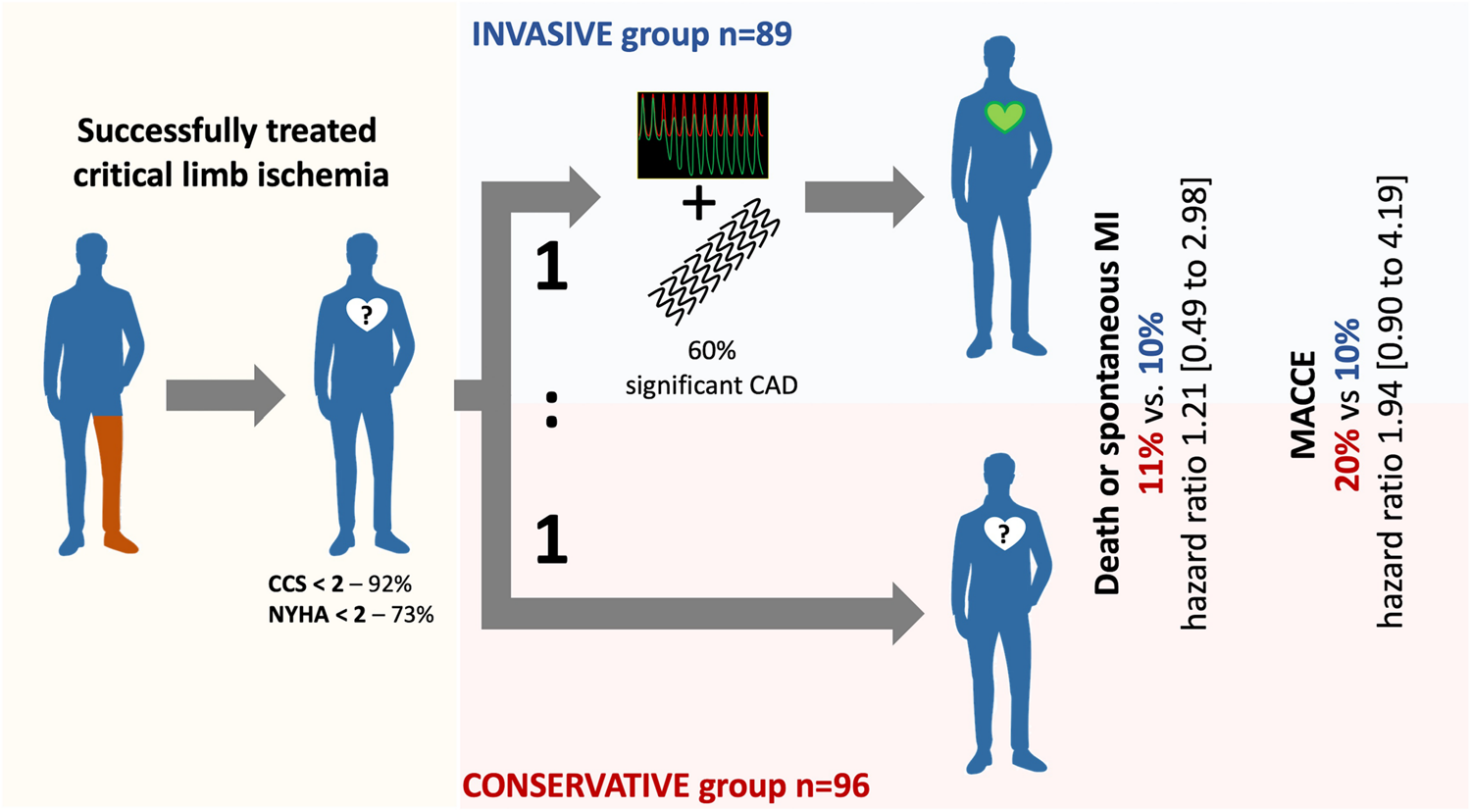

## Introduction

Peripheral artery disease (PAD) is known to be associated with increased cardiovascular risk. Critical limb ischemia (CLI) patients can face up to 20% mortality within 6 months of diagnosis, rising to over 40% at 2 years, primarily due to cardiovascular and cerebrovascular events [1–3].

The mortality rate associated with CLI surpasses that of every other form of occlusive cardiovascular disease, such as coronary artery disease (CAD) due to the systemic atherosclerotic burden. Moreover, non-invasive methods often struggle with diagnostic accuracy in CLI patients due to exercise testing limitations, frequent balanced ischemia, and widespread coronary calcification.

In addition, patients with PAD have poor short- and long-term outcomes even following coronary revascularization compared to those of the overall population. This is likely due to the high occurrence of complex CAD in these individuals [4, 5]. However, when angiography indicates complex coronary pathology without clear functional confirmation, relying solely on angiographic assessment can result in multiple interventions without a definitive ischemic target. Up to 39% of angiographically obstructive coronary lesions lack functional significance. This rate could be higher in patients with severe micro- and macrovascular atherosclerosis, making revascularization of non-ischemic myocardium potentially futile. In contrast, negative effects could arise from unnecessary interventions, which may confound the outcomes [6].

In a prospective registry, 58% of patients treated for CLI showed significant CAD upon coronary angiography (CA). Although this did not influence their 1-year clinical outcomes, however, the functional relevance of the CAD was not considered in treatment strategies [7]. Still, considering the above-detailed limitations of non-invasive methods and recent guideline recommendations, invasive physiologic interrogation may be essential for an accurate risk assessment in this population [8] [9].

Given the significant cardiovascular risks and high pretest probability of CAD in patients with severe peripheral artery disease, *Intentional Coronary Revascularization Versus Conservative Therapy in Patients Undergoing Peripheral Artery Revascularization Due to Critical Limb Ischemia trial* (INCORPORATE trial) aimed to assess the benefits of a default invasive strategy, targeting ischemic regions with complete coronary revascularization, versus a traditional conservative method in patients successfully treated for CLI via peripheral artery revascularization.

## Methods

### Design

**Fig. 1:**
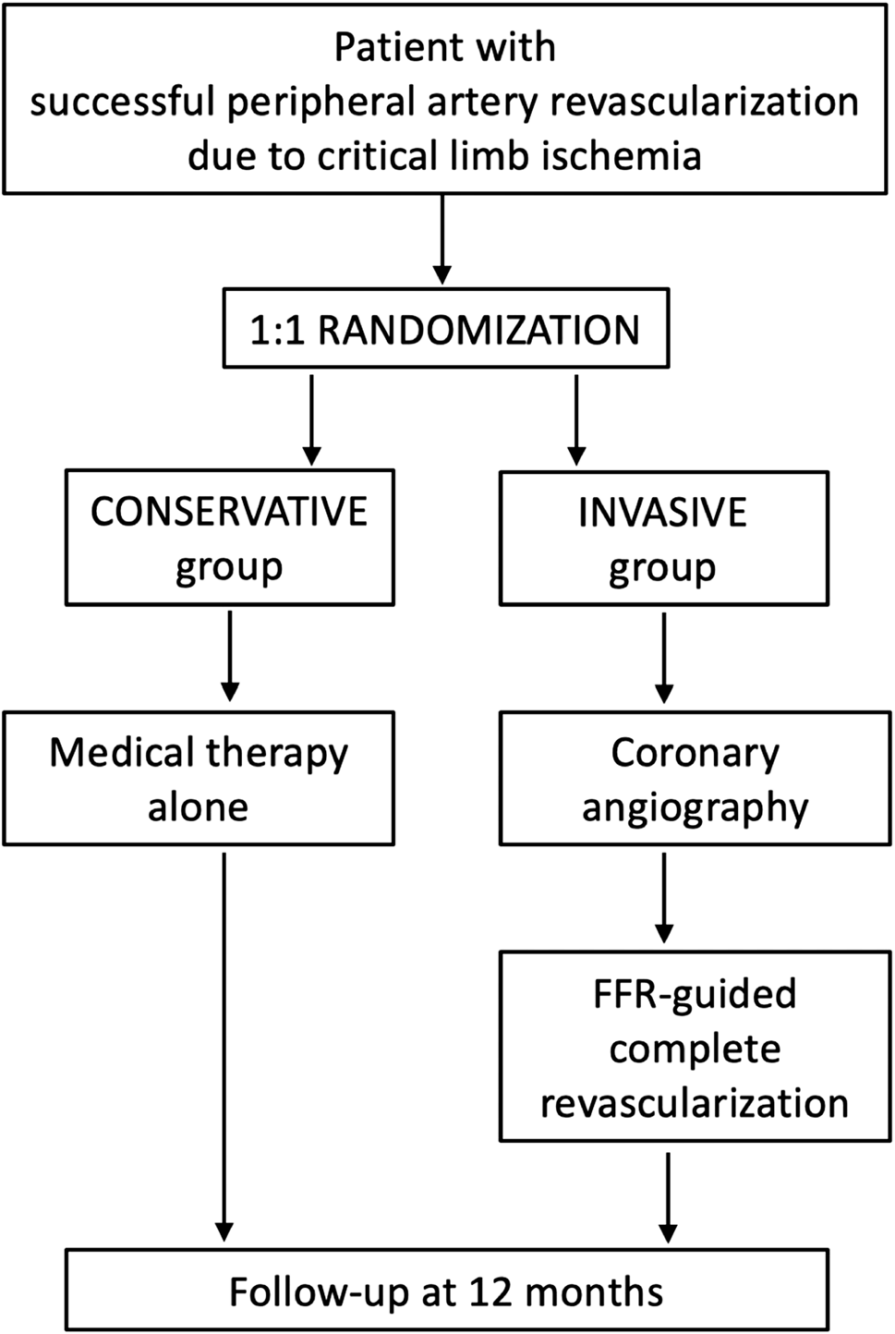
Flow chart of the INCORPORATE trial

The INCORPORATE trial is a prospective, 1:1 randomized, open-label multicentric study across ten cites. The study flow chart is depicted in Fig. 1. The trial aimed to test whether an intentional invasive strategy with ischemia targeted reasonably complete coronary revascularization and optimal medical therapy is superior to a primarily conservative approach with optimal medical therapy alone in patients with treated severe PAD and CLI. The primary endpoint of the trial was the composite of overall death and spontaneous myocardial infarction (MI) at 1-year follow-up. The secondary endpoint was major adverse cardiac and cerebrovascular events (MACCE) at 1 year, as well as the individual elements of the composite endpoint. The detailed design and rationale of the study were published earlier [10]. INCORPORATE is an investigator-initiated trial, partially supported by Boston Scientific (Marlborough, MA, USA) with non-financial support. Protocol is approved by the ethical committees of all collaborating centers. The study was registered at clinicaltrials.gov (NCT03712644).

### Patients

The INCORPORATE trial aimed to enroll 650 patients who had undergone successful percutaneous or surgical peripheral revascularization due to CLI, defined as Rutherford-Becker classification 4 or higher [11]. Patients in the conservative group received optimal medical therapy alone and followed up as per protocol. Any further cardiac investigations were only performed if there was a clinical suspicion of chronic or acute myocardial ischemia-related symptoms. Patients in the invasive group received optimal medical therapy, as well as underwent elective CA, followed by fractional flow reserve (FFR)–guided assessment of existing CAD. Coronary revascularization was performed only if justified by FFR measurements. Patients in both groups did not undergo non-invasive myocardial functional assessments following revascularization of CLI and prior to randomization.

### Statistical methods

In the foundational design of the INCORPORATE trial, as outlined in our previously published paper on the study’s design and rationale [10], our sample size calculation was based on an expected absolute difference of 10% in the composite outcome of spontaneous myocardial infarction and all-cause mortality between treatment groups at 1-year follow-up. This expectation was predicated on an anticipated event rate of 20% in the conservative group and 10% in the invasive group. To achieve 90% power at a 5% alpha level, while accounting for an expected crossover rate of 5%, we calculated a necessary sample size of 650 patients.

All analyses were performed with Prism GraphPad 9.0 (GraphPad Software Inc., CA, USA). Continuous variables are reported as mean ± standard deviation or median [interquartile range], as appropriate. Categorical variables are reported as counts (percentages). The normality of the continuous variables was tested using the D’Agostino-Pearson omnibus normality test. Two-sample *t*-test or Mann–Whitney test was used to compare continuous variables between two groups, and Fisher’s exact or chi-square tests were used to compare categorical variables. Kaplan–Meier method was used to generate time-to-first event curves. Comparison of clinical outcomes between the two groups was performed by Cox regression. The level of significance was set at *p* < 0.05. In the intention-to-treat analysis (ITT), subjects were analyzed based on their initial group assignment, independent of the treatment received, or adherence to protocol. In the per-protocol (PP) analysis, only subjects were included, whose treatment strictly complied with the study protocol. In the as-treated (AT) analysis, patients were categorized on the basis of the treatment strategy they actually received.

## Results

The COVID-19 pandemic significantly impacted the conduct of this clinical trial, causing substantial challenges in patient enrollment, protocol alignment, and data collection, leading to its premature termination. The overall number of patients in the trial was 185, with 96 patients assigned to the conservative group and 89 patients to the invasive group. This was significantly below the projected sample size of 650. The observed event rate in the conservative group was 11%, lower than the anticipated 20%.

Majority of patients were male (67%), and the mean age was 69 ± 9 years. Detailed clinical characteristics are shown in Table 1. Additionally, baseline medications administered to patients in both the invasive and conservative treatment groups, as per their initial randomization in the per-protocol groups, are outlined in Table 2.

**Table 1:** Clinical characteristics

	Conservative group (*n* = 96)		Invasive group (*n* = 89)		*p*
	*n*/mean	% or SD	*n*/mean	% or SD	
Male gender	61	63.5	60	69.8	0.43
Age	70	8.65	68.4	9.4	0.23
Weight (kg)	82	20.7	84	19.8	0.62
Height (cm)	167	19.2	170	18.1	0.17
Hypertension	84	87.5	72	83.7	0.53
Dyslipidemia	66	68.8	57	66.3	0.75
Diabetes mellitus	56	58.3	45	52.3	0.46
Smoking	37	38.5	57	66.3	< 0.01
Family history	8	8.3	19	22.1	0.01
GFR	66	17	65	22	0.69
PCI in medical history	17	17.7	16	18.6	0.99
Myocardial infarction in medical history	14	14.6	10	11.6	0.66
CABG in medical history	4	4.2	7	8.1	0.35
Atrial fibrillation	19	19.8	12	14.0	0.33
Rutherford Score	4.7	0.76	4.5	0.96	0.13
Iliofemoral revascularization	30	31.3	30	31.3	0.64
Femoropopliteal revascularization	22	22.9	22	22.9	0.46
Below-the-knee revascularization	39	40.6	39	40.6	0.55

**Table 2:** Baseline medications administered to patients in both the invasive and conservative treatment groups, as per their initial randomization in the per-protocol groups

Medication	Invasive group		Conservative group		*p*
	*n*/mean	% or SD	*n*/mean	% or SD	
ASA	67	77.9	79	82.3	0.464
Second APT	54	62.8	66	68.8	0.436
Anticoagulant	21	24.4	29	30.2	0.410
Statin	65	75.6	79	82.3	0.279
ACEI/ARB	50	58.1	59	61.5	0.653
BB	44	51.2	47	49.0	0.882
CCB	17	19.8	28	29.2	0.170

Abbreviation used in the table are ASA for aspirin, BB for beta blockers, ALP for antiplatelet drugs, ACEI/ARB for angiotensin-converting enzyme inhibitors/angiotensin II receptor blockers, and CCB for calcium channel blockers

All patients in the study were diagnosed with CLI, with 96% presenting symptoms at or above Rutherford class 4. These cases were successfully treated through various revascularization methods prior to recruitment: 44% underwent iliofemoral, 34% had below-the-knee, and 21% received combined revascularization. Of these interventions, 92.5% involved angioplasty-related procedures, while 7.5% underwent surgical procedures. Specifically, among those treated with angioplasty-related procedures, 35.8% received percutaneous transluminal angioplasty, 32.4% underwent plain old balloon angioplasty, 16.9% were treated with drug-eluting balloons (DEB), and 14.8% had a combination of stent placement and DEB. At baseline, 21.8% of all patients were on aspirin alone, and 78.1% were on dual antiplatelet therapy (DAPT). Within the invasive group, 68.2% were on DAPT and 15.1% were on aspirin alone.

At baseline, cardiac symptoms were predominantly mild and infrequent. Ninety-two percent of patients had a Canadian Cardiovascular Society (CCS) grading of angina pectoris of < 2, while 73% were classified with a New York Heart Association (NYHA) score of < 2.

Of the 89 patients randomized to the invasive group, 73 underwent coronary angiography (CA) as per protocol. Invasive CA was performed during the same hospital stay or within 14 days at the latest. For complex or multi-vessel diseases, complete revascularization could be achieved through multiple staged procedures. There were 16 instances of crossover from the invasive group to the conservative group, primarily due to bed shortages during the COVID-19 pandemic and patient preferences. Angiographically, 81% demonstrated significant CAD: 33% with single-vessel disease and 48% with multi-vessel disease. After functional interrogation by FFR, this was downgraded, yet 60% still had a diagnosis of functionally significant CAD. This comprised 34% with functionally significant single-vessel disease and 26% with functionally significant multi-vessel disease. The average FFR was 0.65 ± 0.12. After the invasive strategy was performed, 91% of the randomized patients were free of remaining functionally relevant stenosis. We observed a protocol deviation in 9% of eligible patients with functionally significant CAD. The decision to not proceed with revascularization in these cases was influenced by multiple factors, including the complexity of the coronary lesions, patient-specific risk factors, and the clinical judgment of the treating physicians. Coronary status and means of revascularization are detailed in Table 2.

**Fig. 2:**
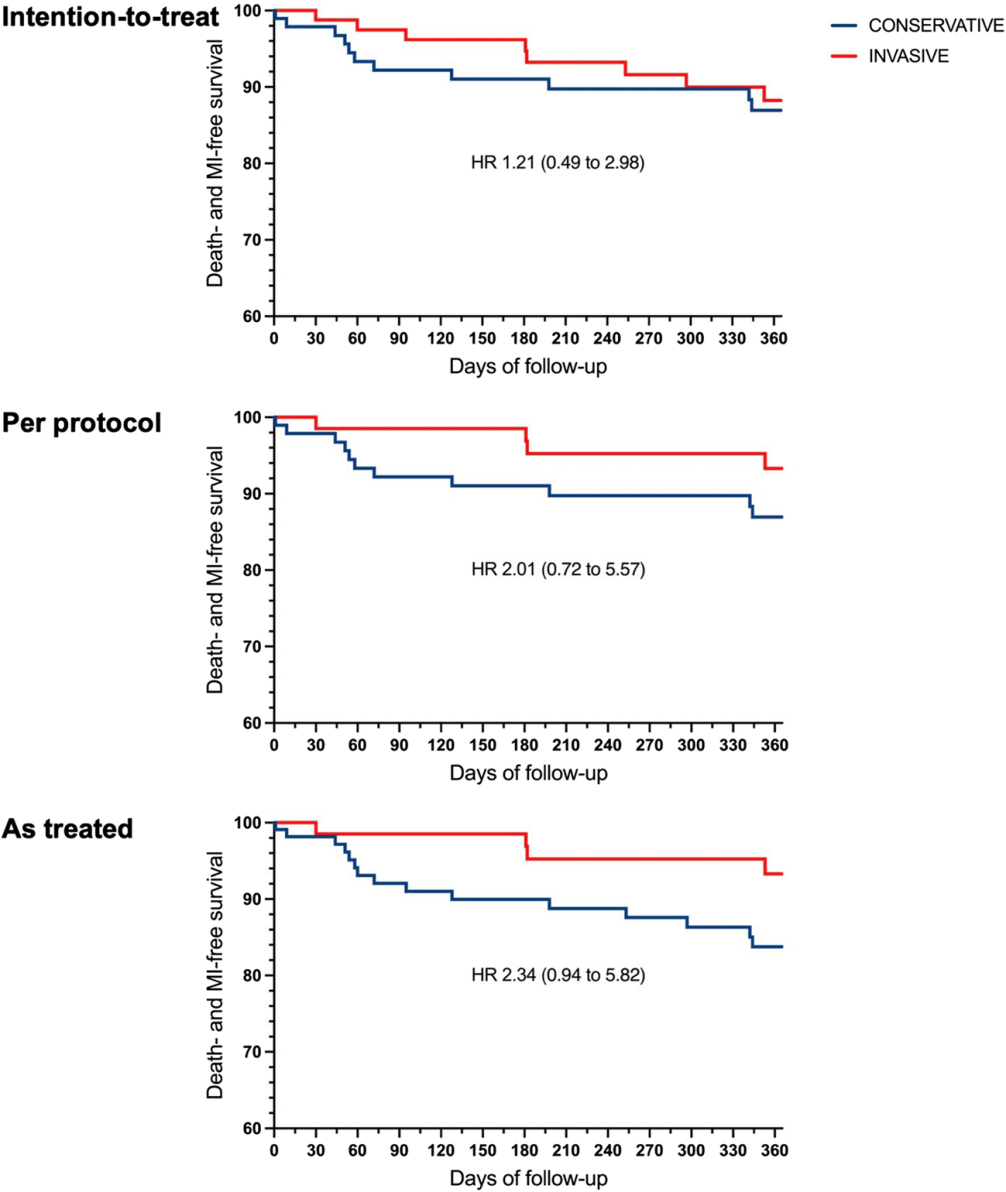
Primary outcome of patients at 12 months according to intention-to-treat, per-protocol, and as-treated analyses

All patients completed 1-year follow-up. In the ITT analysis, the incidence of the combined primary endpoint of death and spontaneous MI at 1 year was similar between the conservative and invasive groups (11% vs 10%; hazard ratio 1.21 [0.49 to 2.98]). Nevertheless, a numerical rise in the risk of MACCE was observed with the conservative approach (20 vs 10%; hazard ratio 1.94 [0.90 to 4.19]). In the PP analysis, the primary endpoint remained non-significant (11% vs 7%; hazard ratio 2.01 [0.72 to 5.57]), while an increased risk of MACCE was shown for the conservative approach (20% vs 7%; hazard ratio 2.88 [1.24 to 6.68]). The primary contributor to this increase in MACCE was a higher incidence of revascularization in the conservative arm. Specifically, these events were driven by urgent revascularizations in five cases, while three cases were attributed to spontaneous myocardial MIs. All revascularization procedures were performed via PCI, targeting the culprit lesion and aiming for a functionally guided complete revascularization whenever possible. AT analysis showed a tendency for a higher incidence of the primary endpoint for conservatively treated patients (14% vs 6%; hazard ratio 2.34 [0.94 to 6.67]) and significant risk of MACCE as well (22% vs 7%; hazard ratio 3.01 [1.38 to 6.56]) (Table 3; Fig. 2).

**Table 3:** Coronary angiographic and procedural characteristics

Invasive group—coronary characteristics		
	*n*	%
Angiographically significant involvement		
Single-vessel disease	24	33%
Multi-vessel disease	35	48%
Left main	7	9%
Left anterior descending artery	44	60%
Left circumflex artery	28	38%
Right coronary artery	29	40%
Functionally significant involvement		
Single-vessel disease	25	34%
Multi-vessel disease	20	27%
Left main	3	5%
Left anterior descending artery	31	42%
Left circumflex artery	12	16%
Right coronary artery	21	29%
FFR—overall	0.77 ± 0.15	
FFR—for functionally significant stenoses	0.65 ± 0.12	
Strategy		
Revascularization performed	39	54%
Single-vessel revascularization	21	29%
Multi-vessel revascularization	18	25%
No functionally relevant stenoses left behind	66	91%

## Discussion

Considering the distinct cardiovascular risk in patients presenting with CLI, the INCORPORATE trial sought to determine whether a proactive approach with default CA and ischemia-targeted revascularization is superior to a conventional conservative approach in terms of spontaneous MI and overall survival at 12 months. The trial was prematurely halted due to COVID-19-related burdens, and therefore, it failed to show significant differences between the outcomes of two groups. Nevertheless, there was a signal of trends towards an unfavorable outcome in conservatively treated patients, especially for the combined secondary endpoint of MACCE (Table 4).

**Table 4:** Primary and secondary outcomes of patients at 12 months according to intention-to-treat, per-protocol, and as-treated analyses

	Intention-to-treat			Per-protocol			As-treated		
	Conservative	Invasive		Conservative	Invasive		Conservative	Invasive	
	*N* (%)	*N* (%)	HR (90% CI)	*N* (%)	*N* (%)	HR (90% CI)	*N* (%)	*N* (%)	HR (90% CI)
Death and spontaneous myocardial infarct	11	8	1.21 (0.49 to 2.98)	11	4	2.01 (0.72 to 5.57)	15	4	2.34 (0.94 to 6.67)
Death	9	8	0.96 (0.37 to 2.49)	9	4	1.66 (0.55 to 4.97)	13	4	2.07 (0.79 to 5.43)
Spontaneous myocardial infarct	3	0	6.67 (0.69 to 64.48)	3	0	6.06 (0.61 to 58.65)	3	0	5.57 (0.56 to 55.29)
Revascularization	8	0	6.33 (1.58 to 25.47)	8	0	5.82 (1.44 to 23.56)	8	0	5.48 (1.34 to 22.39)
Stroke	3	0	5.99 (0.61 to 58.47)	3	0	5.63 (0.57 to 55.70)	3	0	5.40 (0.54 to 54.04)
MACE	16	8	1.74 (0.78 to 3.88)	16	4	2.66 (1.10 to 6.43)	20	4	2.84 (1.26 to 6.38)
MACCE	18	8	1.94 (0.90 to 4.19)	18	4	2.88 (1.24 to 6.68)	22	4	3.01 (1.38 to 6.56)

Given the results of recent trials like ORBITA, ISCHEMIA, and REVIVED, there is a rising debate about the necessity of coronary revascularization in chronic coronary syndrome [12–14]. Contrarily, other studies have demonstrated that when revascularization is limited to patients with extensive ischemia and to lesions which are responsible for it, then beneficial outcomes can be expected compared to pure optimal medical therapy [15, 16]. Even more importantly, patients with significant non-revascularized coronary artery disease were exposed to a greater long-term risk of death or myocardial infarction, even though they presented with no or only mild symptoms at baseline [17].

Actually, the patient population investigated in the INCORPORATE trial shares many similarities with this latter cohort: these patients bear a significant atherosclerotic burden in the peripheral vasculature, which is in itself associated with a higher incidence of coronary atherosclerosis and pronounced cardiovascular risk [18, 19]. This aligns with our findings as well, where almost two-thirds of the patients in the invasive group did indeed have one or more functionally relevant coronary artery stenoses with FFR as low as 0.65 ± 0.12. Notably, the incidence of left main stem and left anterior descending artery stenosis was as high as 45%.

Still, clinical symptoms such as angina or shortness of breath during exercise might remain concealed, which can be explained by the minimal exercise capacity of these patients due to their primary disease. Patients, recruited in the trial, were suffering from Rutherford 4 or more symptoms, suggesting severe claudication or even rest pain in the lower extremities. This suggests that in daily life, these patients rarely engage in exercise intense enough to reach the ischemic threshold of their myocardium. In line with this, we found that fewer than one-tenth of the patients experienced CCS II or worse angina, and only one-quarter had a NYHA II or worse symptomatic status. However, the applicability and interpretability of the CCS and NYHA classification systems for these patients may be questionable. It is important to emphasize, while these patients have minimal or no symptoms, and probably no myocardial perfusion deficiency at baseline, this condition might change dramatically once patients undergo successful peripheral revascularization, releasing the former exercise capacity burden. As exercise capacity becomes less limited by peripheral vascular conditions, the myocardium faces an increase in workload that coronary perfusion potentially cannot comply with. In the best-case scenario, this situation leads to progressive stable symptoms only. However, this might also explain the high incidence of early myocardial infarction and cardiac mortality as the first presentation in patients who underwent treatment for CLI [1, 20–22].

This mechanism suggests that careful cardiologic assessment towards existing CAD might be reasonable for patients, who underwent treatment of CLI. The decision whether to begin with non-invasive tests or to proceed directly with invasive CA remains a topic of open discussion. This choice may also be influenced by local logistical factors. Systematic non-invasive testing as a gatekeeper could have its strategic importance. However, modalities such as ergometry, myocardial scintigraphy, and coronary computed tomography have their own limitations, either in sensitivity or specificity, when dealing with this potentially complex CAD population [23]. However, and as highlighted in a recent consensus paper, particularly with the latest generation of CT scanners, CCTA may possess the capability to not only diagnose but also guide the treatment of CAD, including multi-vessel disease [24]. Stress echocardiography or stress magnetic resonance tomography could offer a more conclusive approach, especially in experienced hands. The results can even guide revascularization, once indicated. On the other hand, one could argue that the threshold for invasive CA could be kept low, considering the inherently high pretest probability. Still, the cornerstone of revascularization strategies has to remain the functional confirmation of lesion significance. This can probably be done equally well by either angiogram-based or wire-based technologies. In the INCORPORATE trial, we specifically avoided non-invasive functional assessments in this high-risk cohort for several reasons. As detailed in the design paper [10], patients with CLI present unique challenges: (1) the inability of exercise testing due to limited physical capacity, (2) frequent occurrence of balanced ischemia, and (3) widespread coronary calcification that limits the diagnostic accuracy of non-invasive tests. Balanced ischemia occurs when there is diffuse and symmetric reduction in blood flow to the myocardium, making it difficult for non-invasive imaging tests to detect regional differences in perfusion. These limitations justify, in our opinion, the use of invasive coronary angiography and FFR measurement to obtain precise functional information on coronary lesions. This approach aligns with the ESC guidelines’ recommendation that when non-invasive tests are not available or inconclusive, invasive measures such as FFR are warranted to guide revascularization decisions. According to the 2019 ESC guidelines, these techniques are recommended for initial diagnosis, especially in patients with lower probabilities of significant CAD. However, invasive coronary angiography remains the gold standard for definitive diagnosis, particularly in high-risk patients with inconclusive non-invasive test results [8].

It is also important to note that since the inception of the INCORPORATE trial, there have been significant advancements in high-quality CT technology and CT-derived fractional flow reserve (CT-FFR). These advancements may warrant consideration in future studies or clinical practice for similar patient cohorts.

While the INCORPORATE trial did not specifically address the impact of routine coronary angiography prior to PAD surgery on surgical risk, it is important to note that existing evidence suggests no significant benefit in this context [25]. Previous studies have indicated that routine coronary angiography before PAD surgery does not reduce surgical risk or improve outcomes. This underscores the need for careful patient selection and highlights the distinct approach of our study, which focuses on FFR-guided revascularization in patients who have already undergone successful peripheral revascularization.

It is noteworthy that only 75% of patients in the intervention group, despite being high-risk, were on statin therapy. This suboptimal use of statins highlights the need for improved adherence to guideline-recommended therapies in high-risk populations, ensuring that all eligible patients receive appropriate lipid-lowering treatment could potentially enhance clinical outcomes. Patients treated invasively were more frequently smokers compared to those in the control group. Smoking is a known risk factor for adverse cardiovascular events, which can influence outcomes. This disparity could have mitigated the potential benefits of revascularization, despite the intervention.

The study has limitations to be declared. The COVID-19 pandemic significantly impacted the execution of the study: It posed substantial challenges in patient recruitment and maintaining protocol adherence for individuals, particularly when rapid discharge was a general policy due to limited hospital capacities. This primarily resulted in a certain rate of crossover, which ultimately exceeded the initially expected rate. Secondly, these issues prompted us to terminate the trial early because of a drastic slowdown in patient recruitment, making it unrealistic to complete the trial within a reasonable timeframe. Thirdly, even if this patient cohort suffers dramatically bad cardiovascular outcome, we observed markedly lower event rate than expected from former literature. Still, we are convinced that the data, which has been generated, despite their limitations, are valuable for enhancing our understanding of this extremely vulnerable patient cohort.

As highlighted in the “Results” section, the observed event rate in the conservative group was notably lower than anticipated, at 11% compared to the expected 20%. This, coupled with the reduced enrollment of only 185 patients, which is 28% of our initial target, has substantially affected our trial’s power. Recalculations indicate that the power to detect a significant difference between groups under these new conditions is approximately 5.57%, markedly lower than the intended 90%. Moreover, the required sample size to achieve the original power, given the observed event rate, would be estimated to be around 39,476 patients, far exceeding our initial projection of 650. This significant disparity highlights the challenges in detecting true differences with such a reduced sample and altered event rates. However, the smaller sample size does raise concerns about the variability and accuracy of the observed event rate. These factors underscore the need for a cautious interpretation of our results. Additional data or studies would be necessary to validate these findings and better understand the true event rates in a larger and more diverse population.

## Conclusions

INCORPORATE demonstrates that in patients with CLI, significant CAD is common, yet cardiac symptoms are scarce. There was no significant difference in the primary endpoint between upfront revascularization and conservative treatment. However, a trend towards higher MACCE in the conservative arm was noted. Therefore, routine cardiac investigation could frequently reveal functionally relevant coronary stenoses, highlighting a potential target to improve outcomes in this vulnerable cohort.
