# Correction: Intentional coronary revascularization versus conservative therapy in patients after peripheral artery revascularization due to critical limb ischemia: the INCORPORATE trial

**DOI:** 10.1007/s00392-024-02501-7

**Published:** 2024-07-24

**Authors:** Gabor G Toth, Marianne Brodmann, Sadeek Sidney Kanoun Schnur, Stanislaw Bartus, Mislav Vrsalovic, Oleg Krestianinov, Petr Kala, Jacek Bil, Robert Gil, Jan Kanovsky, Luigi Di Serafino, Luca Paolucci, Emanuele Barbato, Fabio Mangiacapra, Zoltan Ruzsa

**Affiliations:** 1 Department of Cardiology, University Heart Center Graz, Medical University Graz, Graz, Austria; 2 Division of Angiology, Department of Internal Medicine, Medical University Graz, Graz, Austria; 3 Department of Cardiology, Faculty of Medicine, Doctoral School of Clinical Medicine, University of Szeged, Szeged, Hungary; 4 Royal Cornwall Hospitals NHS Trust, Truro, UK; 5 II Dept of Cardiology, Medical College, Jagiellonian University, Krakow, Poland; 6 Department of Cardiology, University of Zagreb School of Medicine, Sestre Milosrdnice University Hospital Center, Zagreb, Croatia; 7 E. Meshalkin National Medical Research Center of the Ministry of Health of the Russian Federation, Novosibirsk, Russia; 8 University Hospital Brno and Medical Faculty of Masaryk University, Brno, Czech Republic; 9 Department of Invasive Cardiology, Centre of Postgraduate Medical Education, Warsaw, Poland; 10 National Medical Institute of the Internal Affairs and Administration Ministry, Warsaw, Poland; 11 Department of Advanced Biomedical Sciences, University of Naples Federico II, Naples, Italy; 12 Department of Medicine and Surgery, Research Unit of Cardiovascular Science, Università Campus Bio-Medico Di Roma and Fondazione Policlinico Universitario Campus Bio-Medico, Rome, Italy; 13 Department of Clinical and Molecular Medicine, Sapienza University of Rome, Rome, Italy

**Correction: Clinical Research in Cardiology** 10.1007/s00392-024-02487-2

After the article had been published, the 8th author informed us that he is not only affiliated with “9) Department of Invasive Cardiology, Centre of Postgraduate Medical Education, Warsaw, Poland” but also with “10) National Medical Institute of the Internal Afairs and Administration Ministry, Warsaw, Poland”.

The Original article has been corrected.

